# Magnetogenetics: remote non-invasive magnetic activation of neuronal activity with a magnetoreceptor

**DOI:** 10.1007/s11434-015-0902-0

**Published:** 2015-09-14

**Authors:** Xiaoyang Long, Jing Ye, Di Zhao, Sheng-Jia Zhang

**Affiliations:** School of Life Sciences, Tsinghua University, Beijing, 100084 China; School of Medicine, Tsinghua University, Beijing, 100084 China; IDG/McGovern Institute for Brain Research, Tsinghua University, Beijing, 100084 China; Tsinghua-Peking Center for Life Sciences, Tsinghua University, Beijing, 100084 China

**Keywords:** Magnetogenetics, Optogenetics, Iron-sulfur cluster assembly protein, Magnetoreceptor, Non-invasive and remote activation, Neuronal activity and circuit

## Abstract

Current neuromodulation techniques such as optogenetics and deep-brain stimulation are transforming basic and translational neuroscience. These two neuromodulation approaches are, however, invasive since surgical implantation of an optical fiber or wire electrode is required. Here, we have invented a non-invasive magnetogenetics that combines the genetic targeting of a magnetoreceptor with remote magnetic stimulation. The non-invasive activation of neurons was achieved by neuronal expression of an exogenous magnetoreceptor, an iron-sulfur cluster assembly protein 1 (Isca1). In HEK-293 cells and cultured hippocampal neurons expressing this magnetoreceptor, application of an external magnetic field resulted in membrane depolarization and calcium influx in a reproducible and reversible manner, as indicated by the ultrasensitive fluorescent calcium indicator GCaMP6s. Moreover, the magnetogenetic control of neuronal activity might be dependent on the direction of the magnetic field and exhibits on-response and off-response patterns for the external magnetic field applied. The activation of this magnetoreceptor can depolarize neurons and elicit trains of action potentials, which can be triggered repetitively with a remote magnetic field in whole-cell patch-clamp recording. In transgenic *Caenorhabditis elegans* expressing this magnetoreceptor in *myo*-*3*-specific muscle cells or *mec*-*4*-specific neurons, application of the external magnetic field triggered muscle contraction and withdrawal behavior of the worms, indicative of magnet-dependent activation of muscle cells and touch receptor neurons, respectively. The advantages of magnetogenetics over optogenetics are its exclusive non-invasive, deep penetration, long-term continuous dosing, unlimited accessibility, spatial uniformity and relative safety. Like optogenetics that has gone through decade-long improvements, magnetogenetics, with continuous modification and maturation, will reshape the current landscape of neuromodulation toolboxes and will have a broad range of applications to basic and translational neuroscience as well as other biological sciences. We envision a new age of magnetogenetics is coming.

**Electronic supplementary material:**

The online version of this article (doi:10.1007/s11434-015-0902-0) contains supplementary material, which is available to authorized users.

## Abstract (zh)

现有的神经调控技术, 如光遗传学和深部脑刺激, 正在改变基础研究和临床转化神经科学。然而这两种调控方式都是损伤性的, 因为需要在大脑里植入光纤或者金属电极。在这个研究中, 我们新发明了一种非损伤性的神经调控方法, 称之为“磁遗传学”。这个方法结合基因打靶和磁场刺激, 将这个进化上高度保守的外源性的磁感应受体, 一种铁硫蛋白 (Isca1), 导入到大脑特定的区域, 使其在特定的神经元中表达, 然后通过外部磁场刺激, 激活大脑特定的区域。在体外培养的HEK-293细胞和海马神经元中表达该磁感应受体, 施加外部磁场, 能激活细胞, 导致细胞膜去极化和钙离子内流。这种钙离子内流可以由超灵敏荧光钙指示剂GCaMP6s检测出来, 当细胞内钙离子浓度升高时, GCaMP6s的荧光强度会升高。此外, 磁场控制神经元的活动可能依赖于磁场的方向以及磁场的打开和关闭, 对于不同方向的磁场刺激, 以及磁场的开关, 神经元有不同的反应模式。全细胞膜片钳记录的神经元的电信号显示外界磁场可以使表达磁感应受体的神经元去极化, 从而引起动作电位。通过肌肉细胞特定的启动子*myo-3*将这个受体导入线虫的肌肉细胞内, 外加磁场可以触发线虫肌肉收缩 ; 通过神经细胞的特定启动子*mec-4*将磁感应受体导入应力感受神经元, 外界磁场的刺激可诱发线虫后退的行为。磁遗传学和光遗传学相比, 明显的优势是非损伤性, 高穿透深度, 长久持续刺激, 无空间限制性, 空间均匀性和相对安全性。正如光遗传学通过了十年之久的改之后所取得的进展一样, 磁遗传学开辟了一个全新的神经调控领域, 经过持续的发展和成熟, 将有广泛的应用前景, 并将极大地推动基础研究, 转化神经科学以及其它生物科学的发展。我们预见一个磁遗传学时代的来临。

## Introduction

The complex neural microcircuits are the essential building blocks of how the brain works, but they are entangled with interdependent different cell types, interconnected wiring diagrams and internetworked complicated connectome in vivo [1, 2]. Understanding how neural circuits respond to external stimuli, generate electric firing patterns, process information, compute coding, and orchestrate behavior has, therefore, remained a great challenge for neuroscientists [3]. With continuous development and maturation, many neurotechnological toolboxes [4] including optogenetics [5], chemogenetics [6, 7], deep-brain stimulation [8], and functional magnetic resonance imaging (fMRI) [9] have been proven to play an important role in dissecting, perturbing, and modulating interconnected neural microcircuits in the healthy and diseased brain. Among those well-developed neurotechnological toolboxes, both classical deep-brain stimulation and modern optogenetics make it possible to map, monitor, and manipulate physiological and dysfunctional neural microcircuit activity [9, 10]. However, they all have their own limitations or drawbacks. The classical deep-brain stimulation has been successfully used to treat Parkinson’s disease and other neurological disorders, but its limitations are the necessity of surgical implant of an electrical wire, the lack of spatial selectivity or specificity, as well as its contradictory effect of low-frequency and high-frequency stimulation on neuronal excitation or inhibition, respectively [11]. Even though the most popular optogenetics could spatiotemporally activate or deactivate neural activity with a millisecond precision [12–14] and has rapidly transformed neuroscience, the side effects from opsin expression patterns, laser-induced heating, abnormal ions distribution caused by overexpressed pumps or channels, and/or undesired network homeostasis can make experimental interpretation very difficult [15]. Both optogenetics and deep-brain stimulation have been used to invasively manipulate the neuronal activity of a specific subregion in the intact mammalian brain through a permanently implanted electric wire or optical fiber during the chronic surgery [9, 16, 17]. As a result, there has been a high demand on a new generation of exclusively noninvasive neuroperturbation and neuromodulation toolboxes for the whole brain at both microcircuit and macrocircuit levels.

In this study, we invented a noninvasive technique named as magnetogenetics thereafter, which combines the genetic targeting of a magnetoreceptor with remote magnetic stimulation. The noninvasive activation of neuronal activity was executed through an iron-sulfur assembly protein, iron-sulfur cluster assembly protein 1 (Isca1) [18–20]. We speculate that this iron-containing magnetoreceptor might form as an iron-sulfur cluster that could bind to cellular plasma membrane through either cytoskeletons or filaments [18, 21, 22]. We found that this magnetoreceptor could evoke membrane depolarization and action potentials, generate calcium influx, and trigger neuronal activity in both HEK-293 and cultured primary hippocampal neurons when activated by a remote magnetic field. We then renamed this revolutionarily highly conserved magnetoreceptor as MAR. The successful combination of remote magnetic stimulation and genetic targeting will, therefore, reshape the landscape of currently available neuroperturbation and neuromodulation toolboxes including optogenetics and deep-brain stimulation. This novel technology makes the exclusively noninvasive dissection of complex brain circuitry as well as the modulation of deep-brain regions possible, opening a new door to noninvasive, remote, and magnetic control of neuronal activities in the intact mammalian brains and biological processes in other organisms.

## Methods and materials

### DNA constructs

All plasmids were constructed by standard molecular biology procedures and subsequently verified by double-strand DNA sequencing. GCaMP6s and ASAP1 were from Addgene. The AAV-CAG-MAR-P2A-GCaMP6s and Lenti-CAG-MAR-P2A-GCaMP6s were connected via a 2A peptide (P2A) under the chimeric promoter CAG (a combination of the cytomegalovirus early enhancer element and chicken beta-actin promoter). ASAP1 expression plasmid (pcDNA3.1/Puro-CAG-ASAP1) was from Addgene 52519. The AAV-CAG-MAR-P2A-ASAP1 and Lenti-CAG-MAR-P2A-ASAP1 were created with multiple PCR cloning.

### HEK-293 and transfection

HEK-293 cells were maintained and continuously passaged with high-glucose Dulbecco’s modified Eagle Medium (DMEM, Gibco/BRL) containing fetal bovine serum (FBS, Life Tech). Transfection was performed using either Lipofectamine-2000 (Life Tech) or classical calcium phosphate transfection.

### Primary neuronal culture and transfection

Rat hippocampus were dissected from embryonic day 18 rats, and primary cultured hippocampal neurons were cultured has been described [23, 24]. Transfection was performed using either Lipofectamine-2000 (Life Tech) or classical calcium phosphate transfection at different days of in vitro culture.

### rAAV production

The rAAV vector was pseudotyped with AAV1 capsid [25]. The chimeric rAAV2/1 was prepared by co-transfection of human embryonic kidney cell line HEK-293 prepared from co-transfection using the standard calcium phosphate method along with the adenoviral helper plasmid pHelper (Strategene, CA, USA). Twelve hours after transfection, the DNA/CaCl_2_ mixture was replaced with normal growth medium. After an additional 60 h in culture, the transfected cells were collected and subjected to three times of freeze/thaw. The clear supernatant was then purified using heparin affinity columns (HiTrap Heparin HP, GE Healthcare, and Sweden). The purified rAAV2/1 was concentrated with an Amicon Ultra-4 centrifugal filter 100 K device (Millipore, MA, USA), and the viral titer was determined by real-time quantitative PCR using StepOnePlus Real-Time PCR Systems and TaqMan Universal Master Mix (Applied Biosystems, CA, USA). The titered virus was diluted and titer-matched to 1.0 × 10^12^ viral genomic particles/ml by 1 × phosphate-buffered saline.

### Immunofluorescent

For the immunostaining, HEK-293 and neurons grown on cover slips were rinsed three times for 10 min in 1 × PBS at room temperature and pre-incubated for 2 h in 10 % normal goat serum in PBST (1 × PBS with 0.5 % Triton X-100). All rinses between incubation steps were with PBST [24]. After rinsing, processed cover slips were incubated with different primary antibodies against MAR (Homemade, 1:200), NeuN (Millipore, 1:500), and mCherry (Clontech, 1:500) for 72 h in antibody-blocking buffer at 4 °C. After three times of 15-min washing in 1 × PBST at room temperature, cover slips were incubated in a secondary antibody conjugated with either Alexa Fluor 488 or Cy3, respectively (Jackson ImmunoResearch, West Grove, Pennsylvania, USA, 1:500) for 2 h at room temperature. After intensive rinsing with 1 × PBST, cover slips were mounted onto glass slides, and a cover slip was applied [25].

### Growth and transgenesis of *C. elegans* lines

All *C. elegans* strains were grown and maintained on nematode growth media (NGM) agar plates cultured at 20 °C. The NGM agar plates were seeded with OP50 *Escherichia coli*. Transgenic strains were generated through a standard microinjection into N2 worms according to a standard procedure [26]. Untagged *MAR* in transgene *zdEx12[pmyo*-*3::MAR; pmyo*-*3::gfp]* and *zdEx22[pmec*-*4::MAR; pmec*-*4::gfp; sur*-*5::mCherry]* were injected in N2, yielding strains that carried extrachromosomal arrays ZD24, ZD34, respectively. The plasmids *pmyo*-*3::gfp, pmec*-*4::gfp and sur*-*5::mCherry* were co-injected as markers to make sure those specific cells were successfully inherited with the transgenic array. The certain promoter driven GFP (two strains for *myo*-*3* and *mec*-*4*, see Supplementary Table 1) was used to monitor the expression pattern of MAR. The behavior of *C. elegans* in response to the magnetic stimulation was recorded under bright field illumination.

### Whole-cell clamp recording in cultured hippocampal neurons

Neurons were recorded with Axon MultiClamp 700B amplifier (Axon Instruments, USA) immersed in Tyrode’s solution [12]. The intracellular solution of glass pipettes (resistance in the range of 3–8 MΩ) contained (in mmol/L): 125 potassium gluconate, 0.5 EGTA, 4 magnesium ATP, 5 NaCl, 0.3 sodium GTP, 10 phosphocreatine, and 10 HEPES (pH 7.2 with KOH). In Supplementary Fig. 3 where voltage clamp was made, intracellular solution consisted of (in mmol/L) 125 Cs-gluconate, 4 magnesium ATP, 0.3 sodium GTP, 10 phosphocreatine, 10 HEPES, 0.5 EGTA, 3.5 QX-314, 5 TEA, and 2 CsCl (pH 7.2 with NaOH). Inward and outward currents were recorded while clamping neurons at −70 and 0 mV, respectively [27]. Membrane resistance was measured by injecting a 10-mV step lasting 100 ms in voltage-clamp mode.

### Calcium imaging

Calcium imaging was performed with Olympus BX51WI upright microscopy equipped with a 40X water immersion objective and an Olympus DP-80 CCD [28]. The relative change of fluorescence intensity (Δ*F*/*F*0) was extracted using ImageJ. Heat map was generated using MATALB (MathWorks, USA).

## Results

### Induction of calcium influx by MAR via a magnetic field in HEK-293

**Fig. 1:**
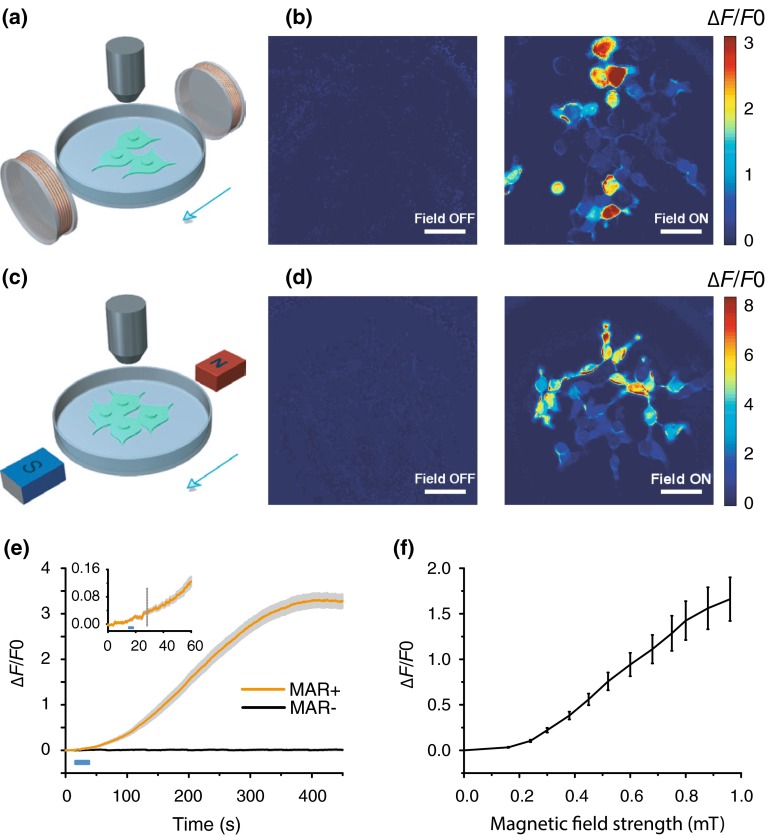
Magnetogenetic activation of HEK-293 cells by remote magnetic stimulation. **a**, **b** Membrane depolarization induced by electric coils. **a** Schematic of magnetic stimulation of MAR-GCaMP6s co-transfected HEK-293 cells by a pair of electrical coils. **b** Heat map showing change of fluorescence intensity (∆*F*/*F*0) before and after magnetic field stimulation. Scale bar, 50 μm. **c–d** Activation of HEK-293 cells with magnetic field generated by a pair of bar magnets. **c** Schematic of magnetic stimulation by a pair of bar magnets. **d** Color map of fluorescence change of GCaMP6s triggered by external magnetic field. Scale bar, 50 μm. **e** Population activity showed increased fluorescence intensity only in MAR-positive cells after magnetic stimulation, while fluorescence intensity of control group remained at the base level. Solid lines, mean; shaded gray areas, s.e.m. Blue bar, field-on. Inset was magnified view showing onset latency of about 13 s after stimulus onset. Dashed line indicated response onset when ∆*F*/*F*0 was 10 folds of the standard deviation of the baseline fluctuation. **f** Quantification of minimum magnetic field intensity needed to elicit response. The average fluorescence intensity of the whole field of view was extracted from a single movie and averaged across 14 different groups of cells. The fluorescence intensity was measured after 27 s of the switch-on of each magnetic field strength. ∆*F*/*F*0 reached 20 % at 0.3 mT

We explored whether MAR could function as a magnetoreceptor and therefore can be used for the magnetogenetic control of neuronal activity with a remote magnetic field. We first co-transfected this MAR (a pigeon homologue of human hIscA) with the genetically encoded and ultrasensitive calcium indicator GCaMP6s [28] into HEK-293 cells, a human embryonic kidney (HEK)-derived cell line. We constructed a custom-made magnetic generator consisting of two pairs of coils, which can hold a standard 35-mm culture dish (Fig. 1a). Our homemade magnetic generator can produce a maximum magnetic field strength of about 1 millitesla (mT) at the center of the dish and approximately 2.5 mT on the edge. Cells at different positions in the culture dish receive different amount of magnetic field strength when stimulated with either our homemade magnetic device or handheld static magnetic bars (Fig. 1c).

**Fig. 2:**
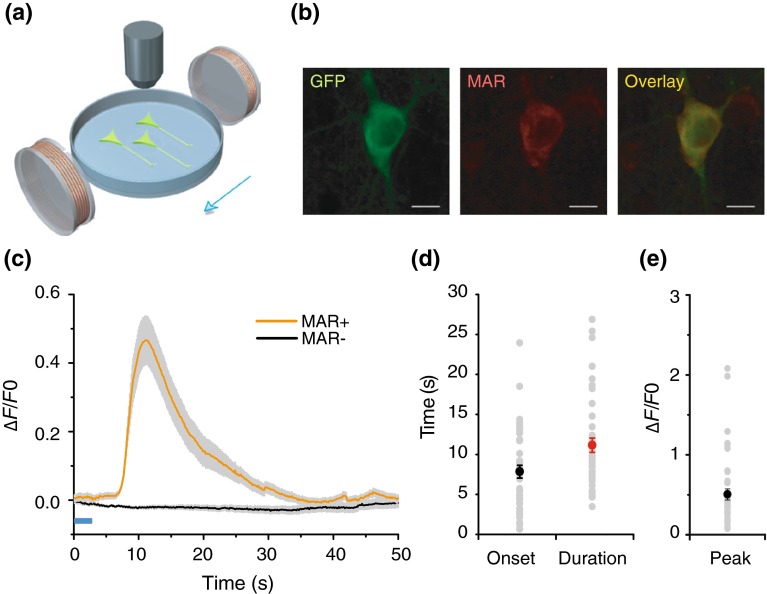
MAR enables magnetic control of neuronal activity. **a** Schematic of calcium imaging with hippocampal neurons cultured in Tyrode’s solution. **b** Confocal imaging showing co-localization of GCaMP6s and MAR. Scale bar, 10 μm. **c** Time course of average peak ∆*F*/*F*0 as a function of time (Solid lines indicate the mean value and shaded gray areas indicate s.e.m.). Calcium transients were only observed in MAR-transfected group. Orange, MAR group, *n* = 42; black, control group, *n* = 40. Blue bar indicates field-on. **d–e** Distribution of onset latency, duration, and peak ∆*F*/*F*0, respectively. Each gray dot represents result from a single neuron, while solid dots indicate mean value. Onset latency was the time interval between the magnetic field onset and the time when ∆*F*/*F*0 reached 10 % of peak ∆*F*/*F*0. Duration was measured between the time when ∆F/F0 increased to 10 % of peak value and the time when ∆*F*/*F*0 decayed to 10 % of peak ∆*F*/*F*0. Mean peak ∆*F*/*F*0 was 50.5 ± 7.0 %; mean onset latency was 7.8 ± 0.8 s; mean duration of MAR-evoked calcium transients was 11.1 ± 0.9 s. Error bar, s.e.m

Before we turned on the magnetic generator, the fluorescence intensity of GCaMP6s in HEK-293 cells remained stable at a base level. After applying the magnetic field, we detected a dramatic increase in fluorescence intensity in MAR-transfected HEK-293 cells (Fig. 1b and Supplementary Video 1), showing almost 350 % increase from about 300 magnet-responsive HEK-293 cells with approximately 94 % of co-transfection rate (data not shown) compared with the base fluorescence intensity (Fig. 1e). The fluorescence intensity kept increasing till the intensity of some of neurons became saturated, implying an underestimate of magnetic field-evoked calcium influx. And some cells in both Fig. 1b and d showed heterogeneous degrees of activation, which may be due to different expression level of MAR, diverse alignment of rod-like MAR within the cells and/or non-uniform distribution of magnetic field strength. This was also the similar case for the magnetic activation of neuronal activity measured shown in Fig. 2. The fluorescence intensity increased to over 10 times of the standard deviation of the base fluorescence intensity, with an average duration of 13 s, indicated by the gray dashed line in the inset of Fig. 1e. Importantly, no increase was observed in control group without the expression of MAR (Fig. 1e). We measured the threshold of magnetic strength by testing the changes of fluorescence intensity in response to magnetic field strength ranging from 0 to 1 mT measured at the center of the culture dish from our homemade device (Fig. 1f). To activate MAR-transfected HEK-293 cells, the minimum magnetic strength required was near 0.3 mT which was about six times higher than the earth’s magnetic strength (~50 μT) [29]. No increase was observed when only the earth’s magnetic field under our working environment was present (data not shown), indicating that the geomagnetic field could not activate MAR and a relative strong magnetic field was needed to elicit response in MAR-transfected cells. Compared to the strong magnetic field strength of up to several Tesla in diagnostic and therapeutic fMRI [9], the magnetic strength present in our study for stimulating MAR was at a level of only several millitesla, suggesting that MAR-dependent magnetogenetic control is not only robust against the influence from geomagnetic field but also safe.

To eliminate the possible artifact due to the background interference from potential fluctuations in the magnetic field generated by the electrical coils of our homemade magnetic generator, we replaced our homemade magnetic generator with handheld static magnetic bars (Fig. 1c) producing almost 2.5 mT at the center of the dish, and found the same observation of dramatic fluorescence increase as that induced by the magnetic generator (Fig. 1d). These observations together suggest that the magnetoreceptor functions as a magnet-responsive activator depolarizes membrane potentials and subsequently generates calcium influx in a magnetic field-dependent way.

### MAR-evoked calcium influx in neuron

We next asked whether MAR can activate neurons and induce calcium influx in MAR-transfected neurons after the application of the external magnetic fields. We co-transfected or infected the primary cultured rat hippocampal neurons using MAR together with GCaMP6s [23, 24] when enriched processes were formed functionally. The immunofluorescent staining showed that MAR appeared to be expressed mainly somato-dendritically (Fig. 2b) with about 71 % of co-transfection rate and close to 90 % of coinfection rate in neuron (data not shown). The MAR-negative neurons showed almost no detectable MAR expression, indicating MAR was produced exogenously not endogenously at least in the hippocampal neurons. Similarly, we could observe the potentiation of Ca^2+^ transients (∆*F*/*F*0 = 50.5 ± 7.0 %, *n* = 42, Fig. 2e) within 7.8 ± 0.8 s (Fig. 2d) after the onset of the externally applied magnetic field (Fig. 2a). Traces were corrected for photobleaching described in Supplementary Fig. 1c. The duration of GCaMP6s in MAR-transfected cultured neurons lasted 11.1 ± 0.9 s (Fig. 2d). As a control, no significant increase in calcium spiking was observed in MAR-negative neurons (*n* = 48, Fig. 2c). We found the minimum magnetic strength required to activate the neurons was similar to that in HEK-293. Furthermore, we could repeatedly activate both MAR-transfected (Supplementary Video 2) and infected (Supplementary Video 3) neurons and detected similar patterns of calcium spike train (Supplementary Fig. 1a, b), suggesting that the magnetic activation of neuronal activity is also quickly reversible. Thus, the magnetogenetic activation of MAR could depolarize neuronal membrane and trigger action potentials quickly and reversibly.

### Magnetic direction-selective control of neuronal activity

**Fig. 3:**
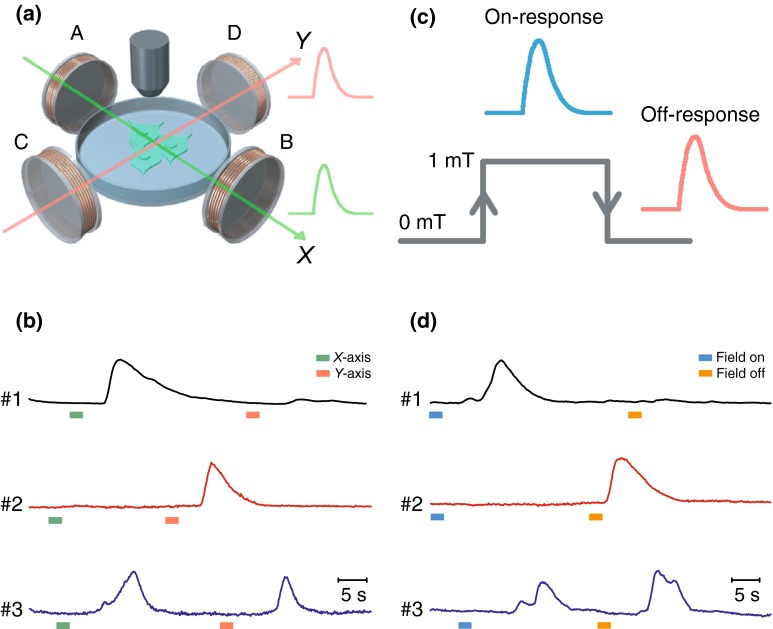
Magnetic direction-selective control and on–off response patterns of neuronal activity. **a** Direction-selective magnetic activation of calcium influx. Schematic of two-directional magnetic stimulation setup. A–B coils produced magnetic field along *X*-direction (green arrow) and C-D coils generated magnetic field along *Y*-direction (red arrow). **b** Sample traces of fluorescence intensity of three neurons in response to magnetic fields of different directions in *X*–*Y* plane. Green arrow, direction of magnetic field in *X*-axis. Orange arrow, direction of magnetic field in *Y*-axis. Left, a representative neuron exhibited a large calcium peak when the magnetic field was turned on to *X*-axis, while only a small peak was observed when the magnetic field was switched to *Y*-axis. Middle, an example neuron responded only to the magnetic stimulation along *Y*-axis. Right, representative trace showing calcium spikes to magnetic field along both *X*-axis and *Y*-axis. Traces showing were ∆*F*/*F*0. Green bar, field on in *X*-direction; orange bar, field on in *Y*-direction. **c** On-response and off-response patterns of neuronal activity. Schematic showing that switch-on and switch-off of magnetic field induced on-response (blue trace) and off-response (red trace) patterns of neuronal activity. **d** Fluorescence traces shown were three representative neurons with different response patterns. Upper, a neuron exhibiting calcium transient when the magnetic field was turned on (on-response), but not when it was turned off (off-response). Middle, a neuron exhibiting off-response but not on-response. Lower, a neuron exhibiting both on-response and off-response. Traces showing were ∆*F*/*F*0. Blue bar, field-on; orange bar, field-off

Since magnetic field has orientation [22], we reasoned that magnetogenetic control of evoked action potentials might be affected by the direction of the external magnetic field applied. To investigate this possibility, we tested the neuronal responses to magnetic fields with different directions. We first checked whether the direction of the applied magnetic field affected the MAR-evoked response of calcium transients of GCaMP6s in our two-dimensional coil-based magnetic generator (Fig. 3a). Since the magnetic field was produced by only one of two pairs of orthogonal coils (A–B and C–D) each time in our homemade magnetic device, we generated magnetic fields along either one of the orthogonal directions, that is, the *X*-direction (from A to B) and the *Y*-direction (from C to D).

We observed that seven out of those 22 magnet-responsive neurons were activated only by magnetic field along the *X*-direction (Fig. 3b, upper panel, Supplementary Video 4), while 11 out of those 22 neurons were activated only by magnetic field along the *Y*-direction (Fig. 3b, middle panel, Supplementary Video 4). Interestingly, the four remaining neurons (4/22) displayed robust calcium spikes in response to both magnetic fields along the *X*-direction and along the *Y*-direction (Fig. 3b, lower panel). We further quantified whether the correlation between the axonal orientation of MAR-transfected neurons and the direction of the applied magnetic field influenced the MAR-triggered responses. No obvious correlation was found between the MAR-triggered response and the axonal orientation relative to the direction of the applied magnetic field (Supplementary Fig. 2a). Since we also found the similar magnetic direction-dependent effect in HEK-293 cells, such directional effect might not be neuron specific, but rather due to rod-like rearrangement of expressed MAR on the cellular membrane under magnetic stimulation. We could not exclude the possibility that expression level of MAR, rod-like cluster redistribution of MAR on the cellular membrane, higher magnetic strength, and/or uniform magnetic activation might eliminate such magnetic direction-dependent heterogeneous effect on neuronal activation. These observations suggested that the magnetogenetic control of action potentials might depend on the direction of the external magnetic field applied in our particular setup given that the maximal magnetic strength cannot exceed 1 mT in our own homemade device. It would be interesting to test the effect of magnetic polarity on neuronal activity with more sophisticated magnetic device in our future experiments.

### On-response and off-response effect of magnetic field on neuronal activity

Since turning the magnetic field on or off might change membrane extension and then open some ion channels in the membrane, we hypothesized that the onset or the offset of the external magnetic field applied could also affect neuronal activity [22]. As expected, we found the on-response, off-response, and on/off-response patterns of neuronal activity when magnetic field is switched on or off (Fig. 3c) in those 22 neurons tested above. We found 12 out of those 22 MAR-GCaMP6s-co-transfected neurons showed dramatic increase in fluorescence intensity when the magnetic field was switched on only. However, the increased calcium transients went back to the base level (Fig. 3d, upper panel, Supplementary Video 5) when the magnetic field was turned off. Interestingly, to the opposite, six out of those 22 MAR-transfected neurons showed no increased activity after the onset of the magnetic field, while GCaMP6s fluorescence showed transient increase when the magnetic field was switched off for the same group of neurons (Fig. 3d, middle panel). Interestingly, a small group of neurons (*n* = 4) responded as actively when the magnetic field was switched from on to off as from off to on (Fig. 3d, lower panel). The distribution of the four different response patterns was summarized in Supplementary Fig. 2b. We could not exclude the possibility that heterogeneous expression of MAR within neurons or rod-like iron-sulfur cluster rearrangement of magnet-stimulated MAR on the cellular membrane and/or non-uniform distribution of magnetic field in our homemade magnetic generator might cause such differential on–off responses of neuronal activity [22]. Future experiments should be performed with a magnetic generator with higher power and more precise control.

### MAR elicits magnetocurrent and spiking in neuron

**Fig. 4:**
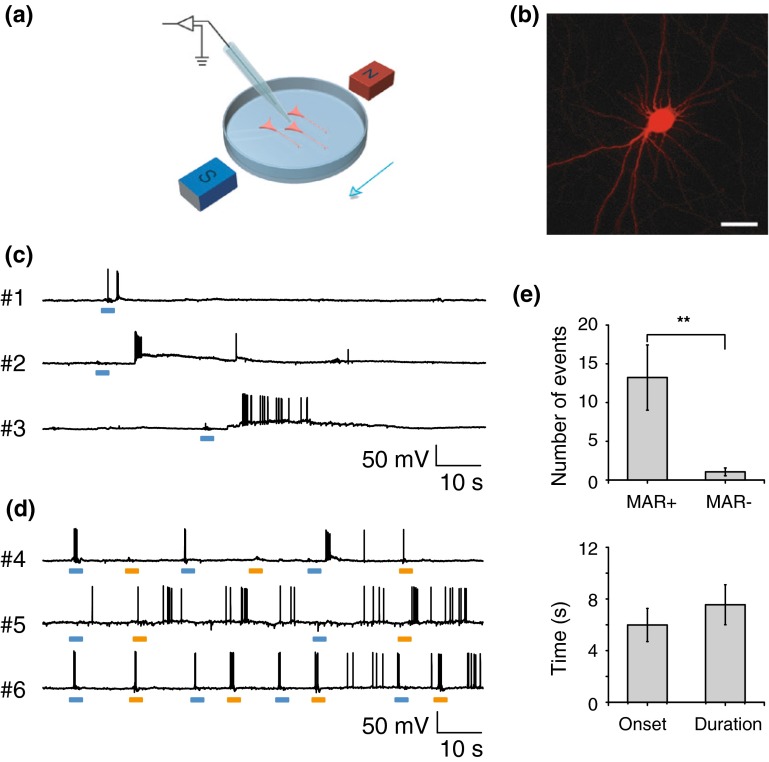
Neuronal spiking activity driven by the magnetic field via MAR. **a** Experiment scheme of whole-cell patch-clamp recording. Magnetic stimulation was achieved through a pair of handheld magnets. **b** Confocal imaging of a typical MAR-p2A-mCherry expressing neuron. Scale bar, 30 μm. **c** Current-clamp recording showing changes of membrane potential to magnetic stimulation. Three example neurons exhibited membrane depolarization and increasing firing rate to the onset of the magnetic field. Scale bar, 10 s, 50 mV. **d** MAR triggered action potentials displayed on-response and off-response firing patterns. Voltage traces of three representative neurons showed distinct firing patterns in response to magnetic field-on and field-off. Upper, the neuron only fired action potentials to the onset of magnetic field. To the opposite, the neuron shown in middle panel mainly responded to the removal of magnet. Another group showed typical firing pattern (lower panel) that both switch-on and switch-off of magnetic field elicited action potentials. Blue bar, field-on; orange bar, field-off. **e** Magnetic field induced significant increase in number of action potentials with mean onset latency of 5.3 ± 1.1 s and average duration of 8.5 ± 1.5 s when compared to spontaneous firing rate (13.2 ± 4.2 spikes versus 1.0 ± 0.5 spikes; *n* = 19; **, *P* < 0.01, paired *t* test). Error bar, s.e.m. Spikes were counted in 20 s after the first elicited spike within 20 s after the magnetic field was turned on

We further examined whether magnet-stimulated MAR can depolarize neurons and evoke a train of action potentials in cultured hippocampal neurons using whole-cell clamp (Fig. 4a) with a pair of handheld static magnetic bars [30], which was used to avoid interference from potential fluctuations in the magnetic field generated by the electrical coils of our homemade device. We transfected neurons with a P2A-linked MAR-mCherry driven by a chicken beta-actin-CMV chimeric promoter [24], ensuring that all identified, mCherry-positive neurons are co-expressed with MAR (Fig. 4b).

Magnetic field evoked rapid inward currents in MAR-positive neurons. Representative recordings showed that whole-cell currents were elicited by application of magnetic field in mCherry-positive neurons clamped at −70 mV (Supplementary Fig. 3a, traces#1–3). Mean inward peak current was 279.6 ± 45.2 pA, and the average number of events was 9.3 ± 3.95 (Supplementary Fig. 3c). Since magnetic field tended to stimulate both excitatory and inhibitory neurons expressing MAR in the culture dish, outward currents could also be recorded in neurons that were voltage clamped at 0 mV [27] (Supplementary Fig. 3b, traces#4–6).

We next investigated whether MAR could drive neuronal firing in a current-clamp mode with the same stimulus used for eliciting magnetocurrent above. Voltage traces shown in Fig. 4c were three representative neurons (traces#1–3) with the increase in firing rate stimulated by external magnetic field. The three neurons exhibited diverse duration of membrane depolarization and different number of action potentials evoked by external magnetic field, which was consistent with heterogeneous activation of neuronal activity revealed by GCaMP6s described above. This implies that gene expression level, alignment of magnetic responsive protein, and/or distribution of magnetic field may contribute to the heterogeneous effects of magnetic field stimulation on the responses of neuronal activity.

Consistent with those results (Fig. 3d) obtained from calcium imaging, we also observed three similar on–off firing patterns stimulated with external magnetic field (Fig. 4d): one activated with on-response only, the second one with off-response only, and the third one with both on-response and off-response. Population data showed that the number of spikes evoked by MAR was significantly higher than spontaneous events (*n* = 19; ** *P* = 0.003, student *t* test), with 13.2 ± 4.2 spikes versus 1.0 ± 0.5 spikes. The spike trains lasted for 8.5 ± 1.5 s with 5.3 ± 1.1 s delay after field onset (Fig. 4e). We quantified the intrinsic electrical properties by injecting a 10-mV voltage step under voltage-clamp mode in MAR-positive and MAR-negative neurons. Both resting membrane potential and membrane resistance showed no significant difference between neurons expressing MAR and those not expressing MAR (Supplementary Fig. 3d). Thus, MAR was able to induce membrane depolarization quickly, evoke action potentials repeatedly, and control neuronal activity remotely.

### MAR can trigger locomotion and induce withdrawal behaviors in *C. elegans*

**Fig. 5:**
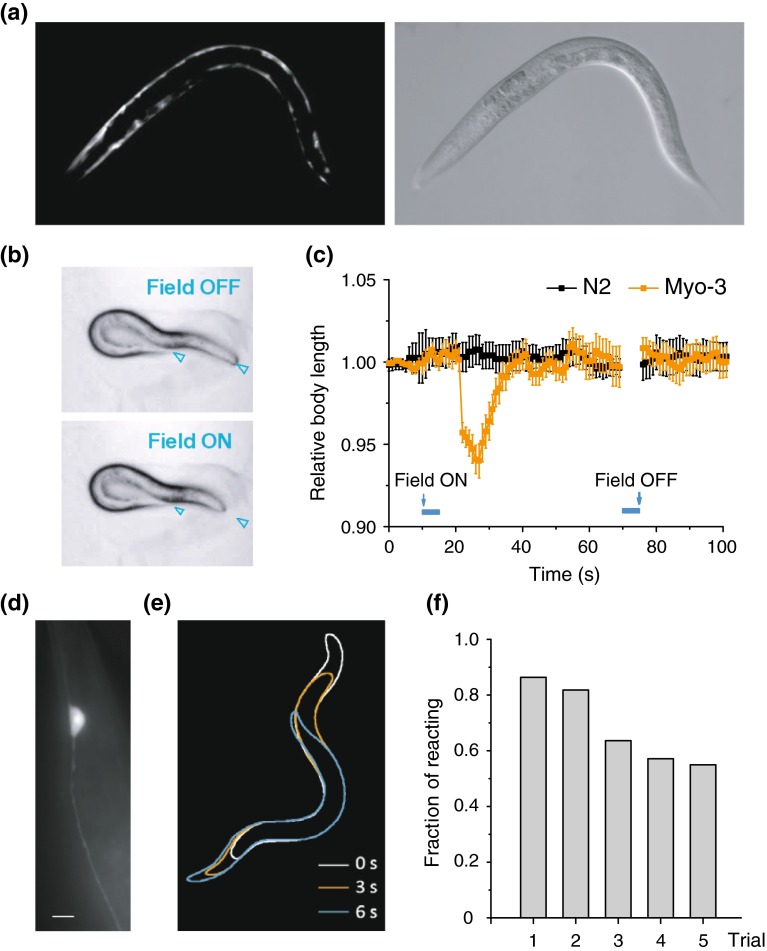
Magnetogenetic control of behavioral responses in *C. elegans*. **a** Epifluorescence image of MAR expression in the body wall of *C. elegans* under the promoter *myo*-*3*. **b** Simultaneous contraction of body muscle when magnetic field was applied under white field illumination. Asterisks indicate the head and tail of *C. elegans*. Left, body relaxation just before magnetic field was on; right, body contraction after the magnetic field was switched on. **c** Body length was measured with 1 s interval at 10 s before and 50 s after magnetic field was turned on and also at 20 s after magnetic field was turned off. Relative body length was calculated by dividing the length measured to the average body length before stimulus onset. Orange trace showing reduction of body length to 94 % of the initial length, while N2 wild type showed no obvious change of body length by magnetic stimulation (*myo*-*3*, *n* = 24; N2, *n* = 20). **d** MAR was selectively expressed in gentle touch receptor neurons under *mec*-*4* promoter. Shown is a PLM neuron. Scale bar, 5 μm. **e** Withdrawal behavior was elicited in the *mec*-*4* transgenic animal when magnetic field was on. Animal positions from 3 frames after stimulus onset at 0, 3, and 6 s were shown by white, orange, blue outline, respectively. **f** Percentage of responding transgenic animals in five consecutive trails with obvious withdrawal or forwarding behavior (with travelling distance of at least 1/4 body length) by magnetic stimulation. All transgenic animals were tested more than five times, and responses were defined as 1 or 0 when the travelling distance met the criteria mentioned above. The fraction of *zdEx22* transgenic *C. elegans* was 86 % in the first trail and showed gradual habituation when tested repeatedly

To test whether the magnet-dependent activation of MAR can trigger circuit and network behaviors in transgenic animals, we constructed transgenic nematode *Caenorhabditis elegans* by expressing MAR under the control of the promoter *myo*-*3*, which restricts its expression to the muscle cells in *C. elegans* [31]. To improve the expression level of MAR in *C. elegans*, we synthesized an artificial MAR gene by optimizing its codon usage, based on its deduced amino acid sequence from pigeon, and by adding two artificial introns that was confirmed to enhance its expression in *C. elegans* [32]. MAR expression was restricted to muscle cells under the promoter of *myo*-*3* (Fig. 5a and Supplementary Fig. 4a).

After applying the external magnet, *zdEx12* transgenic animals displayed robust and reproducible locomotion activity, exhibiting simultaneous contractions of body muscles with apparent shrinkages of the whole-body length on bacteria-fed NGM agar plates (Fig. 5b and Supplementary Video 6).

To quantify the effect of MAR-dependent activation on locomotion [14, 31], we calculated the percentage of body shrinkage. This revealed shrinkages of the body length up to 6 % (Fig. 5c). In contrast, there was no detectable contraction in the wild-type N2 *C. elegans* when the external magnetic fields were applied (*P* < 0.001, paired *t* test). These results demonstrated that MAR can trigger magnet-evoked body contractions or shrinkages of *C. elegans* in vivo.

We next assessed whether magnet-evoked MAR could depolarize neuronal cells and cause subsequent behaviors. We made another *zdEx22* transgenic *C. elegans* in which MAR was selectively expressed only in six mechanosensory neurons AVM, ALML/R, PVM, and PLML/R driven by promoter *mec*-*4* [31]. Figure 5d showed that MAR expression was limited to mechanosensory neurons only under the promoter of *mec*-*4* (see also Supplementary Fig. 4b). MAR triggered withdrawal behaviors in *C. elegans* when the magnetic field was switched on (Fig. 5e and Supplementary Video 7). Nineteen out of 22 (86 %) *zdEx22* transgenic animals showed robust and repeatable withdrawal behaviors under stimulation of magnetic field, in consistent with previous results from ChR2-activated neurons [31]. Remarkably, we observed dramatic omega movement of the whole body of the worm after the external magnetic field was applied (Supplementary Video 8), indicating that unlimited accessibility of the magnetic field could activate all of the six mechanosensory neurons. The same result could not be obtained with optogenetics, which was limited to stimulating only a portion of the six mechanosensory neurons due to the limited penetration depth of light [31]. Occasionally, we could observe accelerations with forwarding behaviors in a few of the transgenic animals. The withdrawal behaviors could be reproducibly evoked by the external magnetic field (Fig. 5f). In contrast, those wild-type control animals did not display withdrawal or acceleration behaviors. Taken together, these results suggest that magnetogenetic control of neuronal activity by MAR could induce behavior output in vivo.

## Discussion

The main discovery of our study is the neurotechnological and conceptual invention of magnetogenetics. The noninvasive magnetogenetics combines the genetic activation of neuronal activity via a magnet-dependent MAR with an external magnetic field, enabling noninvasive and wireless perturbation of neuronal activities with long-term continuous dosing that is almost impossible for optogenetics and pharmacogenetics.

### Nanoparticle-based magnetothermal control of neuromodulation

Anikeeva and her colleagues [33] recently introduced a magnetothermal neuromodulation tool that involved delivering heat-sensitive capsaicin receptor TRPV1 to a particular brain area and then injecting heat-emitting nanoparticles into the same area. This two-step magnetothermal approach has intrinsic drawbacks. First, major safety issues arise from the exogenous Fe_3_O_4_ magnetic nanoparticles permanently incorporated into the brain and from the elevated temperature above 43 °C, well exceeding physiological temperature by heat-emitting magnetic nanoparticles. Second, the diffused magnetic nanoparticles might activate other endogenous thermosensitive ion channels expressed in both peripheral and central nervous systems [34, 35]. Third, since the resonance of magnetic nanoparticles is necessary for producing heat to open TRPV1 channels by alternating magnetic field [33], relative strong magnetic field is desired for neuronal activation (~180 mT versus up to ~2.5 mT in our study).

### The molecular and cellular mechanism of magnetoreception

Vidal-Gadea et al. [36] have recently identified a pair of magnetosensory neurons from *C. elegans* called AFD sensory neurons that respond to geomagnetic field of the earth and support vertical migrations. It remains, however, elusive how AFD sensory neurons detect and use the earth’s magnetic field to guide behaviors. Our finding demonstrates for the first time that a single gene encoding the magnetoreceptor (MAR) could act as a magnetic actuator for controlling neuronal activity. It has been speculated that iron-sulfur assembly proteins with magnet-responsive property might form as magnetosomes and then bind, through either cytoskeletons or filaments, to cellular plasma membrane [21], which is consistent with previously identified genes that are responsible for magnetosome synthesis [37]. After the application of the external magnetic field, the membrane tension due to the magnet-driven rotating force via MAR might cause ion channels to open, thus inducing membrane depolarization and action potential trains [22, 38, 39]. We do not yet know the exact mechanism how the direction of the magnetic field and switching the magnetic field on or off affect the neuronal activity. Further insights could be obtained by studying whether the expression level of MAR, the precise alignment between the three-dimensional magnetic field stimulation and the axon-dendritical orientation of MAR-expressed neurons and/or magnetic strength might affect the direction-dependent magnetic control of neuronal activity [29]. Further studies on MAR-interactive partners and MAR’s own advanced structure might uncover the molecular mechanism for magnetogenetic control of neuronal activity.

### Advantages of magnetogenetics

Our newly invented magnetogenetics has several unique advantages over the decade-long still being optimized optogenetics: Magnetogenetics is noninvasive, remote, penetrative, uniform, and safe. Compared to the optic fiber used in optogenetics [16] and the electric wire assembled in deep-brain stimulation [40], there is no need for chronic surgical implantation of any invasive devices since the external magnetic fields can penetrate deeply into the intact mammalian brain or other biological systems. Although redshifted opsins such as ReaChR [41] and Jaws [42] permit transcranial activation or inhibition of neural activity, respectively, both ReaChR and Jaws can be effective up to only 3 mm deep in the rodent brain [42]. In the meantime, the controllable magnetic field can uniformly act on any central or peripheral nervous systems with precise genetic targeting, overcoming the effect of unevenness due to light absorption and scattering [15]. Furthermore, magnetogenetic stimulation within millitesla range causes no side effects like phototoxicity or thermotoxicity, making magnetogenetics much safer.

### Combination of magnetogenetics with other neuronal readouts

Like all existing genetic and optogenetic activators, silencers, sensors, and effectors [15, 16], this magnetoreceptor uses a single 133-amino-acid-encoded open-reading frame without any cofactor for effective magnetic stimulation. By the use of neuronal cell-type-specific, subregion-specific, or sublayer-specific promoters, delivery of this magnetoreceptor into viral and/or transgenic accessible animals will enable circuit-specific, projection-targeted, and spatiotemporal mapping, manipulation, measurement, and monitoring of neuronal activity in a noninvasive way. A combination of magnetogenetics with genetically encoded calcium indicators and voltage sensors [43, 44], multi-electrode array [45], functional magnetic resonance imaging [46, 47], or multisite single-unit recording [25] will allow us to record large-scale neuronal activity [15, 48] and identify activity patterns corresponding to specific behavioral functions. The application of magnetogenetics will accelerate systematic and causal dissection of neural computation and coding underlying complex interconnected and interdependent brain circuit [2]. Although our study only focuses on magnetic activation by MAR, the opposite way for magnetic inactivation from either a mutated MAR or another undiscovered magnetoreceptor by comparative genomics is feasible. Like direct optogenetic engineering [5], the continuous molecular engineering of diverse families of magnetoreceptors will expand the magnetogenetic toolboxes.

### The application of magnetogenetics to translational neuroscience

Although deep-brain stimulation for treating Parkinson’s disease and other neurological disorders has been proven to be effective, it uses surgically implanted metal electrodes that stimulate targeted regions without any cell-type specificity [10, 40, 49]. While noninvasive transcranial magnetic stimulation (TMS) uses magnetic pulses to induce small electrical currents to stimulate a small region of the cortex [50, 51], its application for basic research and diagnostic and therapeutic use for diseases such as depression and Parkinson’s disease is limited by a lack of specificity, reliability, and replicability. Combined with cell-type-specific promoters [1, 4], magnetogenetics can achieve precisely targeted neuromodulation, overcome non-specificity, and have the potential to benefit therapeutic treatments for Parkinson’s disease as well as other neurological and neuropsychiatric diseases.

### Outlook for magnetogenetics

In summary, noninvasive magnetic activation of neuronal activity with a magnetoreceptor makes magnetogenetics an excellent toolbox for perturbing the activity of complex neural circuitry, enabling the dissection of complex neuronal microcircuitry with cell-type specificity, spatiotemporal precision, spatial uniformity, and noninvasive reversibility. Combined with the genetic targeting of specific cell types and regions, magnetogenetics will accelerate our quest for reaching the ultimate goal of neuroscience: understanding how the brain computes neuronal algorithm, transforms information and generates cognition and behavior. Not only will magnetogenetics have a broad range of applications to basic and translational neuroscience, its principle of using magnetic field for noninvasive, spatiotemporal control of biological systems will also impact other fields in biological science and biomedical engineering [52, 53] at multiple levels including genetic, epigenetic, and transcriptional levels [54]. Like optogenetics with progressive improvement over the past decade, we confidently envision that, with continuous research, development, and optimization, a new age of magnetogenetics is coming in the near future.

## Electronic supplementary material

Supplementary material 1 (DOC 51 kb)

Supplementary material 2 (PDF 598 kb)

Supplementary material 3 (PDF 477 kb)

Supplementary material 4 (PDF 2386 kb)

Supplementary material 5 (PDF 3039 kb)

Supplementary material 6 (MPG 806 kb)

Supplementary material 7 (MPG 930 kb)

Supplementary material 8 (MPG 978 kb)

Supplementary material 9 (MPG 1360 kb)

Supplementary material 10 (MPG 1066 kb)

Supplementary material 11 (MPG 966 kb)

Supplementary material 12 (MPG 766 kb)

Supplementary material 13 (MPG 720 kb)

## References

[1] Luo L, Callaway EM, Svoboda K (2008). Genetic dissection of neural circuits. Neuron.

[2] BRAIN 2025: a scientific vision: brain research through advancing innovative neurotechnologies (BRAIN) working group report to the advisory committee to the director, NIH (2014)

[3] Harris KD, Mrsic-Flogel TD (2013). Cortical connectivity and sensory coding. Nature.

[4] Huang ZJ, Zeng H (2013). Genetic approaches to neural circuits in the mouse. Annu Rev Neurosci.

[5] Zhang F, Vierock J, Yizhar O (2011). The microbial opsin family of optogenetic tools. Cell.

[6] Vardy E, Robinson JE, Li C (2015). A new DREADD facilitates the multiplexed chemogenetic interrogation of behavior. Neuron.

[7] Lerchner W, Xiao C, Nashmi R (2007). Reversible silencing of neuronal excitability in behaving mice by a genetically targeted, ivermectin-gated Cl- channel. Neuron.

[8] Wichmann T, Delong MR (2006). Deep brain stimulation for neurologic and neuropsychiatric disorders. Neuron.

[9] Logothetis NK (2008). What we can do and what we cannot do with fMRI. Nature.

[10] Gradinaru V, Mogri M, Thompson KR (2009). Optical deconstruction of parkinsonian neural circuitry. Science.

[11] Kringelbach ML, Jenkinson N, Owen SL (2007). Translational principles of deep brain stimulation. Nat Rev Neurosci.

[12] Boyden ES, Zhang F, Bamberg E (2005). Millisecond-timescale, genetically targeted optical control of neural activity. Nat Neurosci.

[13] Han X, Boyden ES (2007). Multiple-color optical activation, silencing, and desynchronization of neural activity, with single-spike temporal resolution. PLoS ONE.

[14] Zhang F, Wang LP, Brauner M (2007). Multimodal fast optical interrogation of neural circuitry. Nature.

[15] Häusser M (2014). Optogenetics: the age of light. Nat Methods.

[16] Grosenick L, Marshel JH, Deisseroth K (2015). Closed-loop and activity-guided optogenetic control. Neuron.

[17] Okun MS (2012). Deep-brain stimulation for Parkinson’s disease. N Engl J Med.

[18] Cózar-Castellano I, del Valle Machargo M, Trujillo E (2004). hIscA: a protein implicated in the biogenesis of iron-sulfur clusters. Biochim Biophys Acta.

[19] Mandilaras K, Missirlis F (2012). Genes for iron metabolism influence circadian rhythms in Drosophila melanogaster. Metallomics.

[20] Beinert H, Holm RH, Munck E (1997). Iron-sulfur clusters: nature’s modular, multipurpose structures. Science.

[21] Johnsen S, Lohmann KJ (2005). The physics and neurobiology of magnetoreception. Nat Rev Neurosci.

[22] Winklhofer M (2012). Physiology. An avian magnetometer. Science.

[23] Du J, Feng L, Yang F (2000). Activity- and Ca(2+)-dependent modulation of surface expression of brain-derived neurotrophic factor receptors in hippocampal neurons. J Cell Biol.

[24] Zhang SJ, Zou M, Lu L (2009). Nuclear calcium signaling controls expression of a large gene pool: identification of a gene program for acquired neuroprotection induced by synaptic activity. PLoS Genet.

[25] Zhang SJ, Ye J, Miao C (2013). Optogenetic dissection of entorhinal-hippocampal functional connectivity. Science.

[26] Evans JE, Snow JJ, Gunnarson AL (2006). Functional modulation of IFT kinesins extends the sensory repertoire of ciliated neurons in Caenorhabditis elegans. J Cell Biol.

[27] Jackson MB (2001) Whole-cell voltage clamp recording. Curr Protoc Neurosci Chapter 6:Unit 6 6 10.1002/0471142301.ns0606s00 18428516

[28] Chen TW, Wardill TJ, Sun Y (2013). Ultrasensitive fluorescent proteins for imaging neuronal activity. Nature.

[29] Mouritsen H, Ritz T (2005). Magnetoreception and its use in bird navigation. Curr Opin Neurobiol.

[30] Semm P, Beason RC (1990). Responses to small magnetic variations by the trigeminal system of the bobolink. Brain Res Bull.

[31] Nagel G, Brauner M, Liewald JF (2005). Light activation of channelrhodopsin-2 in excitable cells of *Caenorhabditis elegans* triggers rapid behavioral responses. Curr Biol.

[32] Liu X, Long F, Peng H (2009). Analysis of cell fate from single-cell gene expression profiles in *C. elegans*. Cell.

[33] Chen R, Romero G, Christiansen MG (2015). Wireless magnetothermal deep brain stimulation. Science.

[34] Leibiger IB, Berggren PO (2015). Regulation of glucose homeostasis using radiogenetics and magnetogenetics in mice. Nat Med.

[35] Temel Y, Jahanshahi A (2015). Neuroscience. Treating brain disorders with neuromodulation. Science.

[36] Vidal-Gadea A, Ward K, Beron C et al (2015) Magnetosensitive neurons mediate geomagnetic orientation in *Caenorhabditis elegans*. Elife 4 10.7554/eLife.07493 PMC4525075 26083711

[37] Bazylinski DA, Frankel RB (2004). Magnetosome formation in prokaryotes. Nat Rev Microbiol.

[38] Fleissner G, Stahl B, Thalau P (2007). A novel concept of Fe-mineral-based magnetoreception: histological and physicochemical data from the upper beak of homing pigeons. Naturwissenschaften.

[39] Wu LQ, Dickman JD (2012). Neural correlates of a magnetic sense. Science.

[40] Creed M, Pascoli VJ, Luscher C (2015). Addiction therapy. Refining deep brain stimulation to emulate optogenetic treatment of synaptic pathology. Science.

[41] Lin JY, Knutsen PM, Muller A (2013). ReaChR: a red-shifted variant of channelrhodopsin enables deep transcranial optogenetic excitation. Nat Neurosci.

[42] Chuong AS, Miri ML, Busskamp V (2014). Noninvasive optical inhibition with a red-shifted microbial rhodopsin. Nat Neurosci.

[43] Knöpfel T (2012). Genetically encoded optical indicators for the analysis of neuronal circuits. Nat Rev Neurosci.

[44] St-Pierre F, Marshall JD, Yang Y (2014). High-fidelity optical reporting of neuronal electrical activity with an ultrafast fluorescent voltage sensor. Nat Neurosci.

[45] Spira ME, Hai A (2013). Multi-electrode array technologies for neuroscience and cardiology. Nat Nanotechnol.

[46] Lee JH, Durand R, Gradinaru V (2010). Global and local fMRI signals driven by neurons defined optogenetically by type and wiring. Nature.

[47] Desai M, Kahn I, Knoblich U (2011). Mapping brain networks in awake mice using combined optical neural control and fMRI. J Neurophysiol.

[48] Scanziani M, Häusser M (2009). Electrophysiology in the age of light. Nature.

[49] Benabid AL (2015). Neuroscience: spotlight on deep-brain stimulation. Nature.

[50] Ridding MC, Rothwell JC (2007). Is there a future for therapeutic use of transcranial magnetic stimulation?. Nat Rev Neurosci.

[51] Walsh V, Cowey A (2000). Transcranial magnetic stimulation and cognitive neuroscience. Nat Rev Neurosci.

[52] Stanley SA, Sauer J, Kane RS (2015). Remote regulation of glucose homeostasis in mice using genetically encoded nanoparticles. Nat Med.

[53] Etoc F, Vicario C, Lisse D (2015). Magnetogenetic control of protein gradients inside living cells with high spatial and temporal resolution. Nano Lett.

[54] Cong L, Ran FA, Cox D (2013). Multiplex genome engineering using CRISPR/Cas systems. Science.

