# Supplementary material for: Magnetogenetics: remote non-invasive magnetic activation of neuronal activity with a magnetoreceptor
Source: Sci Bull (Beijing). 2015 Sep 14;60:2107–19. doi: 10.1007/s11434-015-0902-0 (PMC4692962; doi:10.1007/s11434-015-0902-0)
Supplement: Supplementary file 1 — Supplementary material 1 (DOC 51 kb) [file 11434_2015_902_MOESM1_ESM.doc]

**Supplementary Figure 1**

**Calcium influx by repetitive magnetic stimulation in cultured hippocampal neuron.**

**a** Heat map showing change of fluorescence intensity of a representative neuron by repetitive magnetic stimulation. Scale bar, 30 μm. **b** Trace of relative fluorescence change in A by repetitive magnetic stimulation. Blue bars, field-on. **c** Spontaneous fluorescence intensity of a representative neuron was normalized to 1.0 at t=0. Normalized fluorescence was fitted using mono-exponential equation. Traces were then corrected for photobleaching effect with the time constant derived.

**Supplementary Figure 2**

**Summary of angle distribution and fraction of direction-selective responses and on-off responses of magnetic field evoked neuronal activity.**

**a** Quantification of the angle between the axonal orientation of the responsive neuron and the corresponding stimulating direction of the magnetic field. No significant difference was found between the *X*-responsive, *Y*-responsive and both *X*- and *Y*-responsive groups (*P* >0.3, ANOVA test, *n* = 9, 6 and 4, respectively). Error bar, s.d. **b** Fraction distribution of direction-dependent activation and on-off response pattern of neuronal activity by magnetic stimulation.

**Supplementary Figure 3**

**Magnetic field evoked currents and intrinsic properties of MAR-transfected neurons. a** Representative traces showing inward (traces#1-3) currents by clamping neurons at -70 mV. **b** Representative traces showing outward (traces#4-6) currents by clamping neurons at 0 mV. **c** Comparison between magnetic field-evoked inward currents and spontaneous currents. Mean inward peak current evoked by magnetic stimulation was 279.6 ± 45.2 pA versus 33.3 ± 17.8 pA of spontaneous current (***, *P* <0.001, paired *t*-test, *n*=13). The average number of events evoked was 9.3 ± 3.95 vs 0.46 ± 0.24 (**P*<0.05, paired *t*-test). Events were counted in 20 s after the first elicited spike within 20 s after the magnetic field was turned on. **d** Comparison of intrinsic properties between MAR-positive and MAR-negative neurons. Resting membrane potential in MAR expressing neurons (-53.4 ± 3.2 mV, *n* = 14) was not significantly different from neurons not expressing MAR (-52.3 ± 2.4 mV, *n* = 10). (*P* >0.4, *t*-test). Membrane resistance was measured under voltage-clamp mode by injecting a 10 mV voltage step. No statistical difference was found between MAR-positive and MAR -negative neurons (130.6 ± 18.9 MΩ vs 119.8 ± 12.9 MΩ). (*P* >0.3, *t*-test).

**Supplementary Figure 4**

**Epifluorescence image of MAR-expressed muscle cells and mechanosensory neurons. a** Epifluorescence photos showing MAR-localization in the body wall muscle cells indicated by the arrows under the promoter *myo-3* (transgene *zdEx12*). **b** Magnified view of MAR expression in six mechanosensory neurons under the promoter *mec-4 (transgene zdEx22)*. Left, arrows indicate three neurons (AVM, ALMR, PLMR). Right, fluorescent images of the other three neurons (PVM, ALML, PLML).

**Supplementary Table 1.**

***C. elegans*** transgenes and strains used in this study

| Transgene | Genotype | Strain |
| --- | --- | --- |
| *zdEx12[pmyo-3:: MAR; pmyo-3::gfp]* | N2 | ZD24 |
| *zdEx22[pmec-4:: MAR; pmec-4::gfp; sur-5::mCherry]* | N2 | ZD34 |

**Supplementary Video 1.** Calcium imaging of MAR-transfected HEK-293 cells. ‘Field ON’ indicates the application of magnetic field. All calcium imaging shown in supplementary videos 1-5 are 10 times faster than real-time. Scale bar, 50 μm.

**Supplementary Video 2.** Fluorescence recording of MAR-transfected hippocampal neurons. ‘Field ON’ indicates onset of magnetic field. Scale bar, 30 μm.

**Supplementary Video 3.** Calcium imaging of MAR-infected hippocampal neurons. ‘Field ON’ indicates onset of magnetic field. Scale bar, 50 μm.

**Supplementary Video 4.** Calcium imaging showing X-direction and Y-direction magnetic stimulation induces activation of different neurons. ‘Field ON’ indicates onset of magnetic field. Scale bar, 30 μm.

**Supplementary Video 5.** Heat map showing on-response and off-response pattern of one neuron. ‘Field ON’ indicates switch-on of magnetic field; ‘Field OFF’ indicates switch-off of magnetic field. Scale bar, 12 μm.

**Supplementary Video 6.** One transgenic *C. elegans* expressing MAR in body wall muscle cells under the promoter of *myo-3* (transgene zdEx12). The transgenic animal shows simultaneous contractions when the magnetic field is applied (indicated by ‘Field ON’). This supplementary video is 4 times faster than real-time.

**Supplementary Video 7.** One transgenic *C. elegans* selectively expressing MAR in the mechanosensory neurons under the promoter of *mec-4* (transgene zdEx22). The animal exhibits withdrawal behavior when the magnetic field is switched on. This supplementary video is 8 times faster than real-time.

**Supplementary Video 8**. One transgenic *C. elegans* selectively expressing MAR in the mechanosensory neurons exhibits dramatic omega body twist behavior when the magnetic field is switched on. This supplementary video is 10 times faster than real-time.
