# Supplementary figures and images for: Magnetogenetics: remote non-invasive magnetic activation of neuronal activity with a magnetoreceptor
Source: Sci Bull (Beijing). 2015 Sep 14;60:2107–19. doi: 10.1007/s11434-015-0902-0 (PMC4692962; doi:10.1007/s11434-015-0902-0)

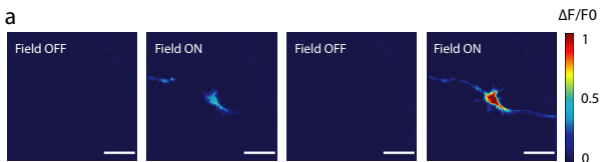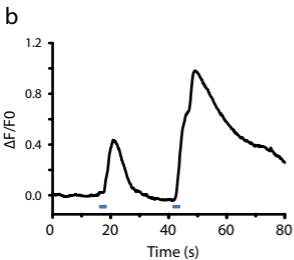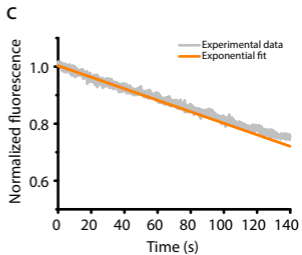

Supplement: Supplementary file 2 — Supplementary material 2 (PDF 598 kb) [file 11434_2015_902_MOESM2_ESM.pdf]

a

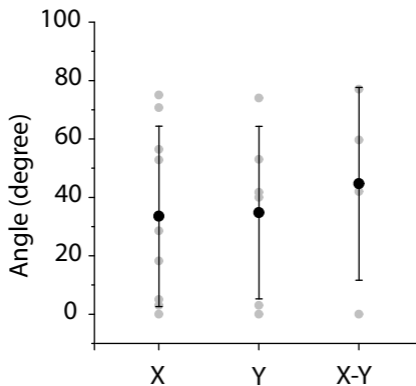

b

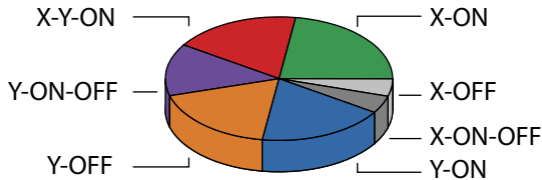

Supplement: Supplementary file 3 — Supplementary material 3 (PDF 477 kb) [file 11434_2015_902_MOESM3_ESM.pdf]

a

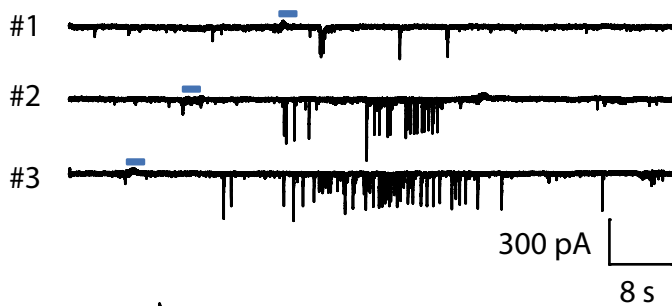

b

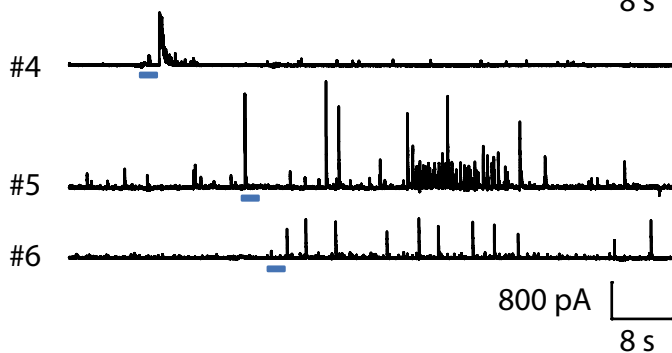

c

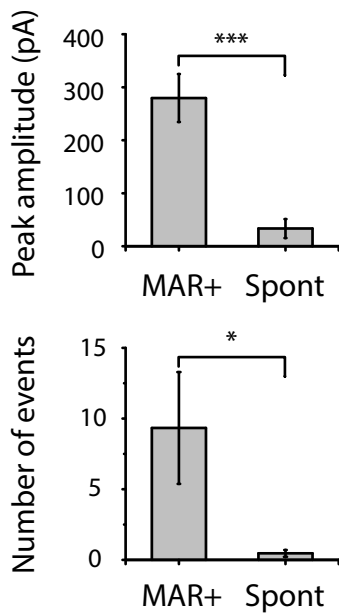

d

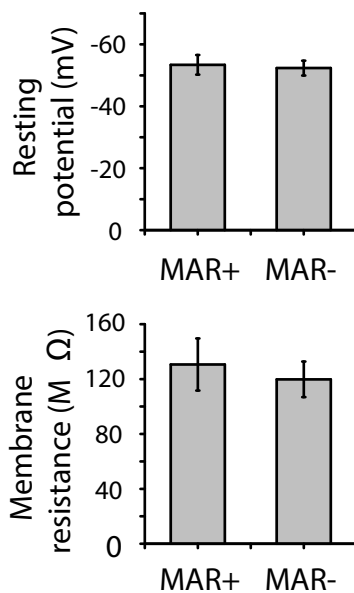

Supplement: Supplementary file 4 — Supplementary material 4 (PDF 2386 kb) [file 11434_2015_902_MOESM4_ESM.pdf]

a

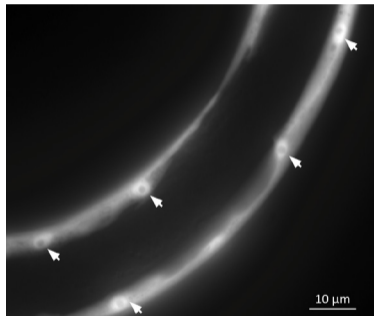

b

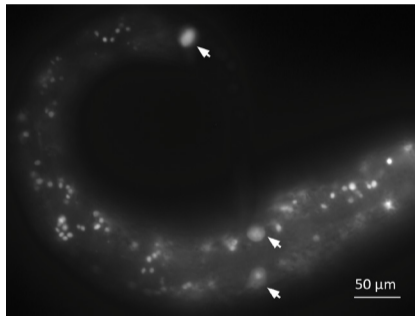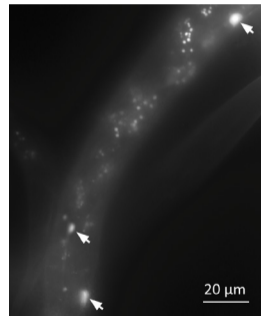

Supplement: Supplementary file 5 — Supplementary material 5 (PDF 3039 kb) [file 11434_2015_902_MOESM5_ESM.pdf]
